# Supplementary material for: Novel Driver Strength Index highlights important cancer genes in TCGA PanCanAtlas patients
Source: PeerJ. 2022 Aug 11;10:e13860. doi: 10.7717/peerj.13860 (PMC9375969; doi:10.7717/peerj.13860)
Supplement: Supplemental Information 2 [file peerj-10-13860-s002.zip › COHORTS/genes plots/cohorts/2021_8_16_14_9_distribution_cohorts_females_KIRC.pdf]

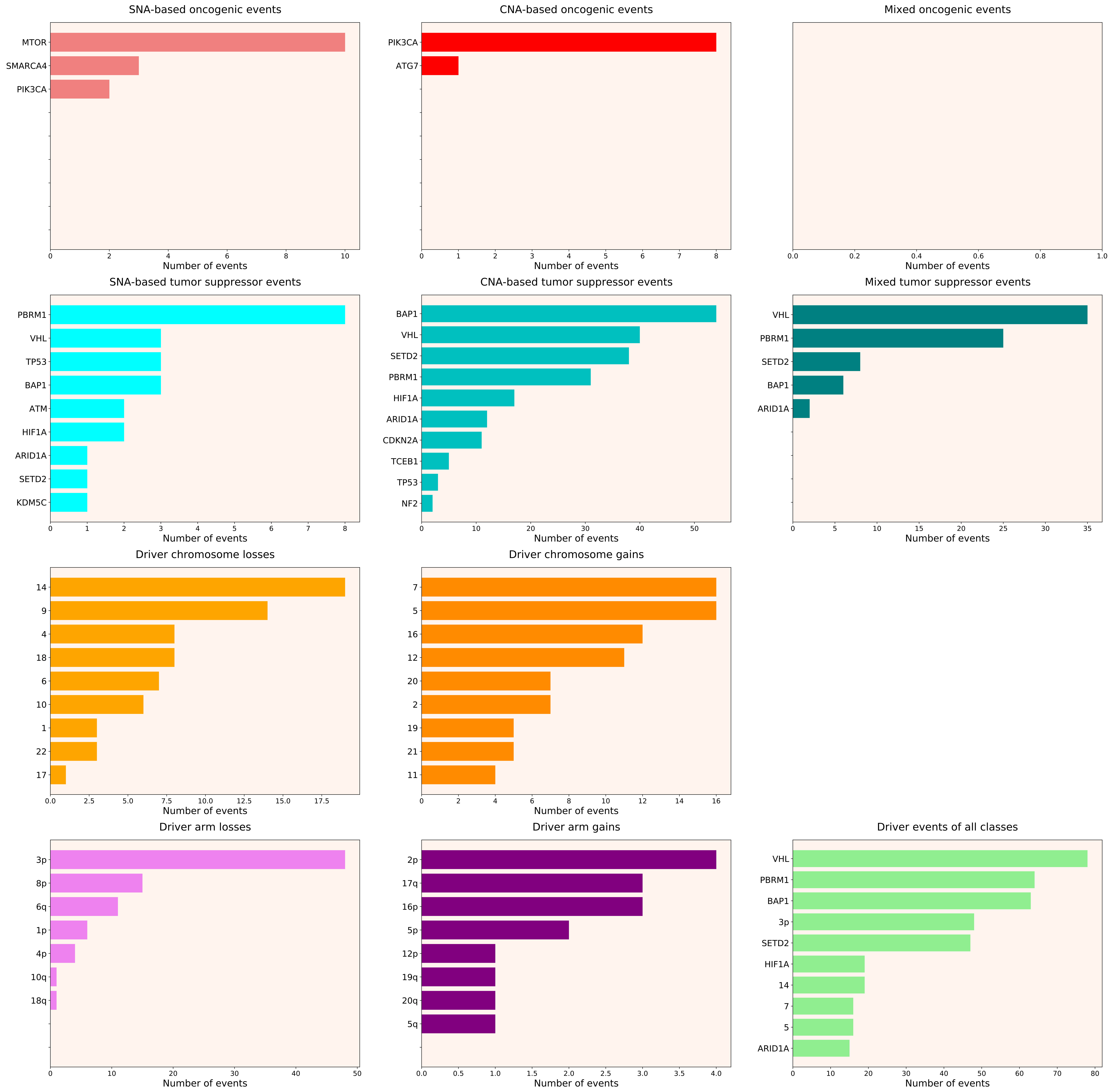

SNA-based oncogenic events
MTOR
SMARCA4
PIK3CA
0 2 4 6 8 10
Number of events
CNA-based oncogenic events
PIK3CA
ATG7
0 1 2 3 4 5 6 7 8
Number of events
Mixed oncogenic events
0.0 0.2 0.4 0.6 0.8 1.0
Number of events
SNA-based tumor suppressor events
PBRM1
VHL
TP53
BAP1
ATM
HIF1A
ARID1A
SETD2
KDM5C
0 1 2 3 4 5 6 7 8
Number of events
CNA-based tumor suppressor events
BAP1
VHL
SETD2
PBRM1
HIF1A
ARID1A
CDKN2A
TCEB1
TP53
NF2
0 10 20 30 40 50
Number of events
Mixed tumor suppressor events
VHL
PBRM1
SETD2
BAP1
ARID1A
0 5 10 15 20 25 30 35
Number of events
Driver chromosome losses
14
9
4
18
6
10
1
22
17
0.0 2.5 5.0 7.5 10.0 12.5 15.0 17.5
Number of events
Driver chromosome gains
7
5
16
12
20
2
19
21
11
0 2 4 6 8 10 12 14 16
Number of events
Driver arm losses
3p
8p
6q
1p
4p
10q
18q
0 10 20 30 40 50
Number of events
Driver arm gains
2p
17q
16p
5p
12p
19q
20q
5q
0.0 0.5 1.0 1.5 2.0 2.5 3.0 3.5 4.0
Number of events
Driver events of all classes
VHL
PBRM1
BAP1
3p
SETD2
HIF1A
14
7
5
ARID1A
0 10 20 30 40 50 60 70 80
Number of events
